# Nutritional training in a humanitarian context: Evidence from a cluster randomized trial

**DOI:** 10.1111/mcn.12973

**Published:** 2020-03-09

**Authors:** Sikandra Kurdi, Jose Luis Figueroa, Hosam Ibrahim

**Affiliations:** ^1^ DSG International Food Policy Research Institute; ^2^ National Institute of Public Health of Mexico

**Keywords:** behaviour change communication, breastfeeding initiation, breastfeeding knowledge, cluster randomized control trial, humanitarian crisis, infant and child nutrition, infant feeding behaviour, programme evaluation, water treatment

## Abstract

Behavioural change communication interventions have been shown to be effective at improving infant and young child nutrition knowledge and practices. However, evidence in humanitarian response contexts is scarce. Using data on secondary outcomes of breastfeeding, water treatment, and knowledge from a cluster randomized control trial of the Yemen Cash for Nutrition programme's impact on child nutritional status, this paper shows that the programme significantly improved knowledge and practices for poor women with young children in the pilot districts. The intervention consisted of cash transfers and monthly group nutrition education sessions led by locally recruited community health volunteers. Data are based on self‐reports by participants. Estimating impacts among all 1,945 women in 190 clusters randomly assigned to treatment versus control and controlling for baseline levels and community characteristic and adjusting for noncompliance with randomization, the programme increased the probability of breastfeeding initiation within the first hour after delivery by 15.6% points (*p* < .05; control = 74.4% and treatment = 83.6%), the probability of exclusive breastfeeding during the first 6 months by 14.4% points (control = 13.5% and treatment = 25.3%), the probability of households treating water consumed by adults by 16.7% points (*p* < .01; control = 13.9% and treatment = 23.4%), and treating water consumed by children under two by 10.3% points (*p* < .10; control = 31.2% and treatment = 37.9%). Impacts on knowledge and breastfeeding are similar for both literate and illiterate women, and water treatment impacts are significantly larger for literate women. This study was registered at 3ie (RIDIE‐STUDY‐ID‐5b4eff881b29a) and funded by the Nordic Trust Fund and Consultative Group on International Agricultural Research programme on Policies, Institutions, and Markets.

**Key messages**

- Nutritional training sessions in the framework of a cash transfer humanitarian response can be effective at changing behaviour.
- Programme design including use of locally recruited women as teachers allowed for equal impact on both literate and illiterate women in breastfeeding knowledge and behaviour
- Suggestive evidence of learning even by women in the community who were not required to attend the sessions

**Abbreviations**
BCC: behaviour change communication
CRT: cluster randomized control trial
EBF: exclusive breastfeeding
IV: instrumental variable
IYCN: infant and young child nutrition
MOPH: Ministry of Population and Health
SFD: Yemen Social Fund for Development
UNICEF: United Nations International Children's Emergency Fund
WHO: World Health Organization
ITT: intention to treat

## 1 INTRODUCTION

In 2016, approximately one in six children were living in areas directly affected by conflict (Kirollos, Anning, Knag Fylkesnes, & Denselow, 2018). Due to the challenges of conducting impact assessments in crisis situations, there has been little rigorous research on the effectiveness of interventions focusing on preventing child malnutrition in such contexts (Blanchet et al., 2017).

Outside of a humanitarian crisis context, there is substantial evidence that behaviour change communication (BCC) can improve infant and young child nutrition (IYCN) knowledge and practices including in developing countries (Bhutta et al., 2008; Imdad, Yakoob, & Bhutta, 2011; Kennedy, Stickland, Kershaw, & Biadgilign, 2018; Saleem, Mahmud, Baig‐Ansari, & Zaidi, 2014; Vazir et al., 2013). BCC specifically regarding promotion of exclusive breastfeeding (EBF) and hygiene education decreases diarrhoeal morbidity (Bhandari et al., 2003; Fewtrell et al., 2005), and there is rich evidence linking improved child‐feeding and hygiene practices to improvements in other health outcomes (Black et al., 2008; Dewey & Adu‐Afarwuah, 2008). BCC interventions are especially effective when they are community based and implemented with awareness of the local cultural context (Bhutta & Lassi, 2010; Britto et al., 2017; Kumar et al., 2018; Sanghvi, Jimerson, Hajeebhoy, Zewale, & Nguyen, 2013; Shi & Zhang, 2011; World Health Organization (WHO), 2008). There is also evidence for the effectiveness of delivering BCC as part of an integrated nutrition‐sensitive intervention including sanitation and food accessibility (Britto et al., 2017; Cumming & Cairncross, 2016; Haselow, Stormer, & Pries, 2016).

Evidence is lacking however on whether community‐based integrated BCC interventions can effectively improve knowledge and feeding behaviours in a humanitarian crisis context (Prudhon, Benelli, Maclaine, Harrigan, & Frize, 2018). In part, due to this lack of a strong evidence base, BCC interventions are often not prioritized in strategies to address child malnutrition in crisis settings (Save the Children, 2012). Using data on secondary outcomes from a randomized control trial of the Yemen Cash for Nutrition programme's impact on child nutritional status, this paper examines a BCC intervention that occurred as part of a nutrition‐sensitive cash transfer intervention in the context of the crisis in Yemen. We show that the program significantly improved participants' practices regarding breastfeeding and water treatment. We also analyse heterogeneity of impact results by mother's literacy, an aspect that has not been widely reported in other evaluations of BCC in spite of awareness that illiterate populations pose distinct challenges (United Nations International Children's Emergency Fund (UNICEF), 2011b). Finally, we provide some suggestive evidence on the existence of positive spillovers to households not enrolled in the programme.

## 2 METHODS

### 2.1 Program description

The civil conflict in Yemen is one of the largest humanitarian crises in the world. Before the war, Yemen already had one of the highest malnutrition prevalence rates across the Arab region, and it is currently estimated that more than 1.8 million children in Yemen are acutely malnourished (UNICEF, WHO, & World Bank, 2018).

The Cash for Nutrition intervention studied in this analysis was a pilot conditional cash transfer programme implemented by the Yemen Social Fund for Development (SFD) in three districts in Al Hodeidah governorate with the aim of reducing the high prevalence of child malnutrition.

The Cash for Nutrition programme targeted female relatives of the mostly male and elderly beneficiaries of the Social Welfare Fund (Yemen's main social protection programme) who had children under 2 years or were pregnant at the time of enrolment in January 2015. Cash for Nutrition participants received monthly cash transfers of 10,000 Yemeni riyals (25% of the value of average monthly food spending) conditional on attendance at monthly nutritional training sessions led by locally recruited community health volunteers. The cash transfers were not labelled for any specific purpose, but most participants reported using them for food, medical expenses, and debt repayment. In practice, the conditionality was not strictly enforced. Rather, the community health volunteers met individually with mothers who were unable to attend the training sessions to find solutions or give individual training sessions.

The community health volunteers each covered several villages and were recruited by the programme from among women living in the targeted area between the ages of 18 and 35 with at least a high school diploma. At the beginning of the intervention, they received 6 days of training in general health education and 6 days of training in nutrition education and measurement of mid‐upper arm circumference for malnutrition screening.

The monthly nutrition and health education sessions for participant women included training on EBF until 6 months, complementary feeding from 6 to 24 months, nutritious meals, handwashing, treatment of drinking water, and how to treat diarrhoea. Additional quarterly sessions targeting pregnant and lactating women covered breastfeeding initiation and the importance of colostrum as well as the consequences of chewing qat and smoking during pregnancy. The manual and visual materials were developed by the Ministry of Population and Health on the basis of messages from UNICEF and WHO localized to the Yemeni context.

In addition to the monthly education sessions, the community health volunteers carried out quarterly screening sessions during home visits to detect and refer cases of malnutrition to health centers for treatment.

The programme was suspended in late 2015 due to the conflict and resumed in the last quarter of 2017, with the result that the beneficiaries in our sample received 9 to 12 months of nutritional training sessions, followed by a month break, and then another 9 months of sessions.

### 2.2 Study design

The outcomes examined here are a subset of those measured in a cluster randomized trial (CRT) study designed to measure the impact of the programme on child nutritional status. As per trial registration, the primary outcome was child height for age, and the infant and young child feeding knowledge and behaviour outcomes examined in this paper were included among the secondary intermediate outcomes. The full study found significant impacts on child height for age for the poorest tercile of households and improvements in dietary diversity and consumption indicators. The study was registered with the 3ie Registry for International Development Impact Evaluations (RIDIE‐STUDY‐ID‐5b4eff881b29a) and was funded by the Nordic Trust Fund managed by the World Bank and the Consultative Group on International Agricultural Research programme on Policies, Institutions, and Markets. A full report on the methodology and all outcomes is available online (Kurdi, Ghorpade, & Ibrahim, 2019).

SFD conducted a preliminary listing identifyng eligible participants for the pilot programme. Because the pilot was not large enough to include all potentially eligible women, SFD divided potential beneficiaries into two priority groups. All women in the first priority group were enrolled, whereas inclusion of women in the second priority group was randomized at the community level. When the pilot was resumed and expanded after the onset of the conflict, this initial randomization for women in the second priority group was maintained by inertia for almost 1 year within the study area due to administrative lags in registering new participants (see Figure 2 for flow diagram of study participants).

**Figure 1:**
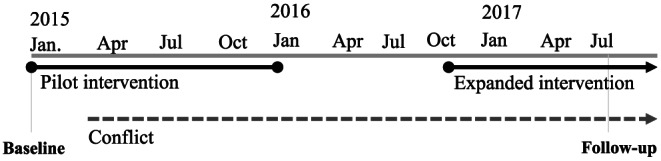
Timeline

A panel household questionnaire in Arabic was used to collect data. The questionnaire topics included household assets, dwelling characteristics, food consumption, education, income sources, and health and nutrition knowledge and practices related to the topics covered in the nutrition and health education sessions. Data were collected by trained enumerators using computer‐assisted personal interviewing on cell phones with the ODK software package. At baseline, the data collection was done through an independent survey organization, Prodigy, and at follow‐up, the data collection was managed directly by the SFD due to the challenges of conducting fieldwork during the conflict. The study included two rounds of data collection. The first round (baseline) was collected in January 2015 prior to the receipt of the first cash transfers. Between baseline and follow‐up, participants received cash transfers and training sessions for 9 months in 2015 before programme suspension and then 12 months in 2016 and 2017. The originally planned follow‐up after 2 years was delayed until August 2017 (2.5 years after baseline) due to security challenges for fieldwork (see timeline in Figure 1).

**Figure 2:**
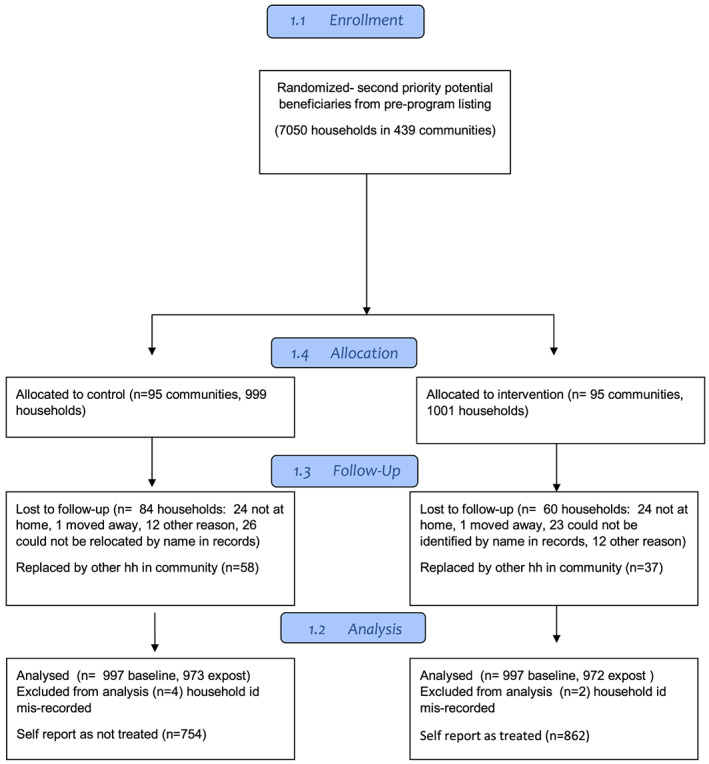
Participant flow diagram

The study was conducted according to the guidelines of the Declaration of Helsinki, and the study received ethical approval from the Institutional Review Board of the International Food Policy Research Institute, Washington, DC. Consent to participate in the study was received verbally after randomization from all respondents included in the survey sample, and this verbal consent was witnessed and formally recorded.

### 2.3 Sample design

The study used a CRT design because of concerns about perceived fairness if randomized at the individual level. Communities used for clustering were defined by SFD in the preliminary listing. Using a random number generator, each community was assigned a random number. Then villages were sorted by strata in ascending numerical order. The first 37% of communities in each strata were assigned to treatment, then the next 37% as control for a total sample size of 95 treatment and 95 control communities. The researchers instructed SFD staff to enroll second priority households in communities selected for treatment and not in communities selected for control. The researchers then similarly randomly selected second priority women in both treatment and control communities to survey.

Power calculations for sample size were estimated for the primary study outcome of child height for age *z* scores. The number of study participants per community ranged from 5 to 30 depending on the number of second priority women available. In total, 2,000 households were included in the study sample; of these, 1,001 were in communities assigned to the treatment group and 999 to the control group. Eighty‐four households from the control group and 66 from the treatment group dropped from the study between baseline and follow up, resulting in 1,850 households surveyed at follow‐up. An additional six households were dropped due to errors in household IDs. Average total attrition rate thus was 7.5% and did not differ significantly between treatment and control. An additional 95 replacement households were added at ex‐post from among eligible households in the randomized clusters that had not been surveyed at baseline. For specific outcomes, the sample analysed was smaller than the total number of surveyed households due to nonresponse to the specific questions in the survey (see sample sizes in Table 1).

**Table 1:** Baseline study sample characteristics for control and treatment groups

	Mean (*SD*)	
	Control	Treated
Household characteristics		
Asset index^a^	0.167 (1.938)	−0.123 (1.779)
Number of people in house	6.537 (3.188)	6.258 (3.548)
Number of children 0–2 years in household	0.813 (0.526)	0.786 (0.542)
Number of children 0–5 years in household	1.728 (0.79)	1.709 (0.792)
Number of rooms	1.306 (0.632)	1.302 (0.586)
Husband in household (%)	0.746 (0.436)	0.729 (0.445)
Households who benefit directly from SWF transfers (%)	0.763 (0.426)	0.723 (0.448)
Households who earn income from employment (%)	0.723 (0.45)	0.743 (0.44)
Households who receive remittances (%)	0.153 (0.36)	0.169 (0.375)
Presence of other food distribution programs (%)	0.179 (0.384)	0.28 (0.449)
Mother characteristics		
Age of mother	28.341 (6.791)	27.754 (6.902)
Age of mother at marriage	17.583 (2.814)	17.541 (2.952)
Mother is illiterate (%)	0.74 (0.439)	0.805 (0.397)
Water treatment		
Treating water for children under two (%)	0.125 (0.331)	0.12 (0.326)
Treating water for adults (%)	0.052 (0.222)	0.033 (0.178)
Nutrition knowledge		
Total knowledge score	9.178 (2.137)	9.063 (2.269)
Knows babies should be breastfed within first hour after birth (%)	0.812 (0.391)	0.769 (0.178)
Knows babies under 6 months should not be given anything but breastmilk (%)	0.554 (0.497)	0.624 (0.485)
Child characteristics (under 2 years old)		
Age of child in months	11.132 (6.871)	11.054 (6.768)
Child is male (%)	0.501 (0.500)	0.521 (0.500)
Infant and young child feeding practices (under 2 years old)		
Baby breastfed during the first hour after birth (%)	0.684 (0.465)	0.645 (0.479)

Abbreviation: SWF, Social Welfare Fund.

Asset index calculated by principal components analysis.

### 2.4 Measures

In this paper, we report on the programme's impact on self‐reported practices of early initiation of breastfeeding, EBF, and water treatment as well as knowledge of the topics covered in the nutritional training sessions. Complementary feeding practices were also examined, but no significant impacts were found.

In terms of practices, we collected data on timing of breastfeeding initiation as a recall question for children under 2 years and complementary feeding and EBF as current practices for children under 6 months old and 7–24 months old, respectively.

Early initiation of breastfeeding is defined as whether the mother breastfed her child within the first hour after delivery. The survey question was asked as a multiple response question and gave the options of within 1 hr, within 6 hr, within 24 hr, within 2 to 3 days, or never. It is common in this context for mothers to give infants sugar and water during the first couple of days after birth as they wait for their milk to come in.

EBF indicates that infants did not receive any foods or liquids but breastmilk. These two outcome variables are measured at the child level. We collected data on EBF and early initiation of breastfeeding at both baseline and follow‐up; however, the baseline question on EBF was asked incorrectly at baseline, so our analysis of EBF relies on the ex‐post data. 1At baseline, the question was asked as “Do you give the baby anything except for breastmilk?,” which prompted a high rate of self‐reported EBF compared with the correct approach of asking whether or not the baby had consumed different items during the past 24 hr for a list of items including water, sugar water, tea, fruit juice, milk, breastmilk, and formula. So we resurveyed a subsample of women (on the basis of accessibility of the villages) several months after the baseline using the standard question format to get a better estimate of baseline prevalence of EBF.


Complementary feeding for children 7 months to 24 years is defined as whether the child is receiving any solid foods.

The water treatment outcomes are based on two separate survey questions in which the woman was asked whether the household treated drinking water consumed by either adults or children under 2 years old. There was no corresponding question in the knowledge module that addressed knowledge about the importance of water treatment.

The outcome for IYCN knowledge is an index specific to this programme constructed based on 13 knowledge questions in the survey that were developed to reflect the content to the nutritional training sessions. The index reflects the number of questions that were answered correctly by interviewees (from 0 to 13). The questions included measure knowledge about (a) location of the nearest health centre, (b) the need to eat more during pregnancy/breastfeeding, (c) the need to drink more during pregnancy/breastfeeding, (d) not using qat during pregnancy/breastfeeding, (e) giving children more water when sick, (f) giving more food to children when sick, (g) sweets being not healthy for children, (h) qat being not healthy for children, (i) malnutrition leading to anaemia, (j) food sources of iron (know at least one), (k) breastfeeding feeding initiation starting within 1 hr after delivery, (l) 6 months being the right age to start complementary feeding, and (m) EBF during the first 6 months. We also separately report impacts on knowledge about breastfeeding initiation and knowledge about EBF for comparison with the impacts on practices.

Data collected on child health outcomes included the primary outcome of height for age as well as weight for age and incidence of diarrhoea in past 2 weeks.

### 2.5 Statistical analysis

The analysis was carried out using the statistical package Stata 15 (StatCorp LP).

We use two different specifications to model the effect of the programme. All models exploit the panel nature of the data by using observations from baseline and follow‐up.

The main specification includes two binary variables: one indicating the intended treatment status of the household at follow‐up and a second one indicating the survey round (1 for *follow‐up* and 0 for *baseline*). The impact of the programme is given by the coefficient associated with the dummy for treatment at follow‐up. This specification also includes household level dummy variables (fixed effects) that eliminate any time invariant household characteristics that differed by random chance between households in the control group and households in the treatment group.

For EBF, we use only the follow‐up data due to the problem with data quality in the original baseline mentioned above. 2A second minisurvey that asked only about exclusive breastfeeding was collected in August 2015 in response to the problem with data quality at baseline, although not all communities could be reached for this survey. An alternative specification with similar results for the impact on EBF using the second minisurvey plus the follow‐up data as a panel is available in Table S1. For both breastfeeding initiation and EBF, we are unable to use household fixed effects because most households did not have a child of breastfeeding age at both rounds of the survey. For these two outcome variables, we instead controlled explicitly for the characteristics that had been found to differ between treatment and control groups at baseline.

In spite of the randomization, some control households benefited from the programme and self‐reported being part of the programme at the follow‐up survey. Self‐reported participation was defined as 1 during the follow‐up survey if the household reported receiving cash transfers in 2016 and 2017 and if they had attended at least one nutritional training session and 0 otherwise. Of the households assigned to control, 220 (23%) reported having been enrolled in the programme, likely due to local programme administration including them by mistake or due to pressure from the household. Of the households assigned to treatment, 89% self‐reported being in the programme. Accordingly, the programme's impact estimation is done using (a) a conservative intention‐to‐treat (ITT) approach, on the basis of the original randomization, and (b) the impact of treatment on the treated by instrumenting self‐reported treatment by original assignment. Use of instrumental variables (IVs) in this way for estimating impact of treatment on the treated is a common approach in economics literature to adjust for noncompliance with randomization (Angrist, 2006; Sussman & Hayward, 2010).

The evaluation was planned to analyse heterogeneity in behavioural change by literacy level because the programme was specifically designed to reach an illiterate population through the use of pictures and role play. For this reason, we also conduct heterogeneity analysis to test if programme impacts were different for literate and illiterate mothers by splitting the sample and comparing the coefficients from the pooled seemingly unrelated regressions using a Wald test.

Standard errors are all adjusted for clustering at the community level.

### 2.6 Ethics considerations

The study was registered with the 3ie Registry for International Development Impact Evaluations (RIDIE‐STUDY‐ID‐5b4eff881b29a) and received ethical approval from IFPRI's Internal Review Board. During the data collection, all participants were asked to provide verbalconsent of participation, and only those who consented wereincluded in the analysis.

## 3 RESULTS

### 3.1 Characteristics of the study sample

We provide household and child baseline characteristics by treatment status in Table 1. The first two columns show information for treated and control households on the basis of the original random assignment. The samples are similar overall with differences likely due to chance rather than any issues with the randomization.

In spite of the soft conditionality, attendance at the nutritional education sessions was high. About 96% of surveyed households in treated communities reported attending at least one training session, and the average number of training sessions attended during the final 12 months of the programme was 8.2 (*SD* = 2.1). Households in control communities who were included in the cash transfer programme due to imperfect compliance with the randomization attended sessions approximately as frequently as households in the treatment communities (mean 8.2 sessions with *SD* = 1.4). Furthermore, households in control communities that were not incentivized to attend the training sessions with the cash transfers also occasionally attended the training sessions with a mean of 0.6 sessions attended.

### 3.2 Effects of the program

Table 2 reports impacts of the Cash for Nutrition programme on breastfeeding practices and treatment of drinking water and correct answers on the knowledge questions. The first column in the second panel of Table 2 shows ITT estimates. The ITT estimates are the simple comparison of the households assigned to treatment with the households assigned to control; however, these estimates understate the true impact of the programme, as the control group include 23% of control household that were enrolled in the programme contrary to the randomization plan. The IV estimates in the bottom panel compare actually treated households with the actually nontreated households using the random assignment to control for self‐selection bias and therefore better capture the full impact of the programme. In our description of the results, we focus on the IV specification. The lower number of observations for EBF and breastfeeding initiation outcomes is due to only collecting responses from households with children under 6 months (for EBF) or under 2 years (for breastfeeding initiation).

**Table 2:** Estimated programme impacts

	Mean values at baseline and follow‐up				Cash for Nutrition programme impacts				Impact estimation details for this outcome			
	Baseline (2015)		Follow‐up (2017)		ITT estimation^a^		IV estimation^b^					
Outcome	Control (*n* hhs/clusters)	Treatment (*n* hhs/clusters)	Control (*n* hhs/clusters)	Treatment (*n* hhs/clusters)	Programme impact [95% CI]	Time trend	Programme impact [95% CI]	Time trend	Fixed effects	Balance controls^c^	Minimum detectable effect^d^	ICC at baseline
Initiation within 1 hr after birth (share of households)	0.676 (797/95)	0.655 (776/95)	0.744 (410/89)	0.836 (452/91)	0.123^**^ [0.028, 0.218]	0.093**	0.156^**^ [0.036, 0.275]	0.07	Village	Yes	0.15	0.099
Child under 6 months EBF; follow‐up only (share of households)	‐	‐	0.135 (89/51)	0.253 (95/56)	0.114^*^ [−0.006, 0.235]	‐	0.144^*^ [−0.009, 0.298]	‐	None	Yes	0.13	0.048 (at ex‐post)
Treating water for adults (share of households)	0.054 (998/95)	0.039 (996/95)	0.139 (916/94)	0.234 (934/95)	0.114^***^ [0.058, 0.170]	0.114***	0.167^***^ [0.085, 0.248]	0.047*	Household	No	0.07	0.135
Treating water for children (under two; share of households)	0.124 (998/95)	0.132 (996/95)	0.312 (916/94)	0.379 (934/95)	0.070*[−0.009, 0.150]	0.070*	0.103^*^ [−0.012, 0.218]	0.164***	Household	No	0.09	0.071
Total knowledge score (mean score out of maximum 13 correct answers)	9.101 (997/95)	9.070 (997/95)	9.401 (915/93)	9.899 (935/95)	0.624^***^ [0.186, 1.063]	0.219	0.913^***^ [0.273, 1.553]	0.000	Household	No	0.55	0.190
Knowledge on early initiation (share of households answering correctly)	0.806 (997/95)	0.771 (997/95)	0.787 (915/93)	0.866 (935/95)	0.121^***^ [0.055, 0.188]	‐0.022	0.177^***^ [0.081, 0.274]	−0.064*	Household	No	0.09	0.079
Knowledge on EBF (share of households answering correctly)	0.560 (997/95)	0.621 (997/95)	0.661 (915/93)	0.776 (935/95)	0.047 [−0.027, 0.120]	0.109***	0.069 [−0.037, 0.174]	0.093***	Household	No	0.09	0.097

Abbreviations: CI, confidence interval; EBF, exclusive breastfeeding; ICC, intra‐cluster correlation; ITT, intention‐to‐treat; IV, instrumental variable.

a Impact of the programme identified using ordinary least squares regression analysis as the coefficient on the interaction between being assigned to treatment and being a response to the ex‐post survey after controlling for time invariant household‐level characteristics and the average time trend.

b Impact of the programme identified using instrumental variables regression analysis as the coefficient on the interaction between the predicted value for reporting as a participant at ex‐post and being a response to the ex‐post survey after controlling for time invariant household level characteristics and the average time trend.

Balance controls for models without household fixed effects are household asset index, number of people in house, husband living in the house, presence of other food distribution programmes in the community, and mother's age, age at first marriage, and literacy.

Minimum detectable difference from ex‐post power calculation at *α* = .05

* *p* < .10.

** *p* < .05.

*** *p* < .01.

In terms of reported practices for mothers, we find a 15.6% (*p* < .05) increase in the probability of reporting breastfeeding initiation within the first hour after delivery (*p* < .05) for children under two at the time of survey. We also find a significant impact on the probability of EBF for women with children under 6 months of 15.6% points (*p* < .1). We did not find any impact of the programme on the age at which children receive solid foods above 6 months (results not shown). The programme led to a 16.7% points increase (*p* < .01) in the probability of treating water consumed by adults. The magnitude of this impact is quite large given the extremely low share of households that reported having treated drinking water for adults at baseline (4% and 5%, for treated and control groups, respectively). Similarly, we observe a significant positive impacts of 10.3% points on the share of households that treated drinking water for children under two (*p* < .1). As with knowledge on EBF, there is also a strong positive time trend on the water treatment outcomes, particularly for treatment of water for children under two of about 16% (*p* < .01). Finally, the programme increased the total knowledge score by 0.91 more questions answered correctly based on the random assignment specification (*p* < .01). For comparison with impacts on practices, we specifically look at knowledge on breastfeeding. The programme increased correct answers on breastfeeding initiation by 17.7% points (*p* < .01). There was no significant impact on correct answers about EBF; however, there is some evidence of a positive time trend showing an increase of 9.3% points (*p* < .01) between baseline and follow‐up.

### 3.3 Heterogeneous effects of the program by maternal literacy

In our sample, 73% of mothers could not read or write (see Table 1), and these women had lower levels of knowledge and correct practices at baseline. Table 3 shows the results for nutrition knowledge and water treatment after splitting the sample by the mothers' literacy status. For the heterogeneity analysis, we report ITT results only to avoid concern about noncompliance rates differing by literacy level. The magnitudes of the coefficient for programme impact on the knowledge outcome variables are actually larger for illiterate women than for literate women; however, a Wald test for the equality of the two coefficients shows that the difference is not significant.

**Table 3:** Estimated programme impacts by mother literacy

	Mean values at baseline and follow up by literacy status				Cash for Nutrition programme impact ITT estimation	Test for equality of impacts by literacy status
Literacy of mother	Control (*n*)	Treatment (*n*)	Control *n*)	Treatment (*n*)	Programme impact [95% CI]	p value from Wald test for equality of coefficients
Treating water for adults						
Illiterate	0.037 (733)	0.039 (794)	0.135 (676)	0.216 (751)	0.085^***^[0.026, 0.143]	0.025**
Literate	0.102 (265)	0.040 (202)	0.150 (240)	0.311 (183)	0.219^***^ [0.126, 0.313]	
Treating water for children (under two)						
Illiterate	0.093 (733)	0.120 (794)	0.288 (676)	0.341 (751)	0.034 [−0.053, 0.121]	0.006***
Literate	0.211 (265)	0.178 (202)	0.379 (240)	0.536 (183)	0.212^***^ [0.104, 0.319]	
Total knowledge score						
Illiterate	8.844 (732)	8.887 (795)	9.135 (675)	9.733 (752)	0.677^***^ [0.179, 1.174]	0.273
Literate	9.811 (265)	9.792 (202)	10.150 (240)	10.585 (183)	0.454 [−0.110, 1.017]	
Knowledge on breastfeeding initiation						
Illiterate	0.783 (732)	0.752 (795)	0.766 (675)	0.850 (752)	0.122^***^ [0.047, 0.197]	0.658
Literate	0.872 (265)	0.847 (202)	0.846 (240)	0.934 (183)	0.118^*^ [−0.002, 0.237]	
Knowledge on EBF						
Illiterate	0.553 (732)	0.613 (795)	0.634 (675)	0.762 (752)	0.061 [−0.017, 0.140]	0.426
Literate	0.577 (265)	0.653 (202)	0.738 (240)	0.836 (183)	0.014 [−0.124, 0.152]	

Abbreviations: CI, confidence interval; EBF, exclusive breastfeeding; ITT, intention‐to‐treat.

* *p* < .10.

** *p* < .05.

*** *p* < .01.

In contrast, for water treatment, the programme impact coefficients are significantly higher for literate women. In terms of treating water for adults, the impact was 21.9% points for literate women compared with only 8.5% points for illiterate women; for treating water for children, 21.2% points for literate women, and no significant impact amongst illiterate women.

The sample size of the EBF outcome was not large enough to split by literacy status.

## 4 DISCUSSION

This paper presents the results of a BCC intervention integrated with cash transfers programme in Yemen between 2015 and 2017. To our knowledge, this is the first paper providing evidence on the effectiveness of IYCN BCC during a civil conflict using a randomized design (Blanchet et al., 2017; Prudhon et al., 2018). The conflict context complicated programme administration, with a break in funding leading to a 1‐year suspension in cash transfers and training sessions. Households were also affected directly by the conflict, with the share of households reporting food insecurity in the past 7 days increasing by 22% points between baseline and follow‐up, and 7% of households in our sample reporting that they were hosting relatives displaced by the conflict (Kurdi et al., 2019).

Our results show that despite these additional challenges related to the conflict environment, the Cash for Nutrition programme was effective in improving women's knowledge and practices. The 11–14% point impact on EBF is quite substantial, considering the initial low levels of observed EBF of 9.2% in a similar sample of Social Welfare Fund beneficiary households in Al Hodeidah governorate in 2011 and unlikeliness of improvement without the intervention during recent period of conflict (UNICEF, 2011a). In the period between baseline and follow‐up, women's dietary diversity declined significantly and there was a 17% point increase in the share of women reporting challenges with producing sufficient breastmilk and a 15% point increase in the use of formula. Although we do not have reliable baseline data on EBF rates, the context was one in which EBF was becoming more difficult, a common concern in humanitarian emergencies. Our impact finding appears modest in magnitude compared with those found in randomized control trials of BCC interventions on EBF in similar but nonconflict settings with low levels of maternal education (Bhandari et al., 2003; Haider, Ashworth, Kabir, & Huttly, 2000). The lower magnitude in our impacts may be related to the fact that EBF was only one component of the nutritional education provided by the Cash for Nutrition intervention rather than the sole focus of the intervention, the strength of traditional beliefs about the need to give infants water and sugar, and the crisis context. The impact on nutritional knowledge of approximately 0.5 standard deviations in the number of correct answers is modest; however, it provides evidence that increase in knowledge was part of the pathway to improving practices. In comparing the impacts, for example, with a recent nonconflict setting intervention in Bangladesh (Hoddinott, Ahmed, Karachiwalla, & Roy, 2018) that finds increase of approximately 1.7 standard deviations in the number of correct answers, it is necessary to consider first that the Cash for Nutrition training component was limited to 1 year of monthly sessions that were interrupted for 9 months due to the civil conflict, in contrast to the 2 years of weekly sessions in Bangladesh. 3The phrasing of the knowledge questions in our survey may also have overstated the baseline level of knowledge as being relatively high (see Figure S1). For example, we know that mothers at baseline did not understand that giving infants water and sugar was a violation of the recommendation for exclusive breastfeeding, and the knowledge question “Should babies under 6 months be given anything except breastmilk? (Yes or No)” did not capture this misunderstanding. Anecdotal evidence from discussion with community health volunteers suggests that the success of the programme in changing behaviour in spite of the difficulties of the setting was related to the fact that the trainings were provided by women from the local community, which allowed a trusting relationship between the participants and the community health volunteers and for the programme to run effectively without the need for strict administrative oversight. These observations would be consistent with evidence about the importance of BCC being well‐integrated into the local community and culture. Some community health volunteers also described how they took seriously the idea that their role was to be a community leader beyond the minimum requirements of the contract with SFD. and this may have been a factor in the programme's ability to impact behaviour in spite of the challenges posed by the conflict setting.

Our impact results are specifically meaningful for guiding humanitarian programming within Yemen. Given the high levels of child mortality in Yemen even before the conflict and the elevated risks of lack of access to safe water during the current crisis, these changes in breastfeeding practices have the potential to be significant for child health outcomes in Yemen (Edmond et al., 2006; UNICEF, 2018; United Nations Office for the Coordination of Humanitarian Affairs, 2018). These results on water treatment are also especially critical in the Yemeni context due to the recent outbreaks of cholera in the country that affects more than 360,000 Yemenis (WHO, 2017).

The results also provide insights about the programme's effectiveness for illiterate women. Formal schooling has been shown to interact with nutrition knowledge in terms of the relationship with child health outcomes, plausibly because mothers with higher education are better able to assimilate and apply health messages that they have received (Webb & Block, 2004); however, well‐designed context‐specific BCC should be able to use appropriate materials and methods to reach illiterate populations (WHO, 2008). The fact that we find no difference in impacts on knowledge by literacy level, but do find differences on practices, is consistent with this literature. Community health volunteers from the programme anecdotally confirmed that they felt it took more time for the less educated women in their groups to reach a level of understanding for putting the knowledge into practice. This difference in impacts on water treatment by literacy status may also be related to the fact that the programme's visual educational materials related to water treatment were not as numerous as those related to breastfeeding or the fact that some forms of water treatment, unlike breastfeeding, involves direct costs, and literate women were more likely to be from households with the resources to apply this knowledge. The most common forms of water treatment in our sample were boiling (with costs related to use of fuel) or drinking only bottled water. However, we do find significant albeit smaller positive impacts on water treatment for adults among illiterate women, which points to the success of the programme design in partially overcoming this challenge.

Finally, the paper provides suggestive evidence of knowledge spillover effects. We find a positive trend between 2015 and 2017 in the probability of treating water and breastfeeding initiation for the control group (the fact that this shows up even in the IV specification shows that it is not just a result of imperfect compliance), showing that outcomes improved even for nonparticipants in the programme who were living in communities where other women (in the first priority group) were participating. The positive trend in these outcomes is notable given the overall negative trend in all economic indicators between baseline and follow‐up due to the conflict. 4The positive time trends on these variables could be due to increasing awareness on avoiding contaminated water that could lead to cholera contagion, as well as positive spillover effects from transmission of information from community health volunteers to nonparticipating households. In a community‐level survey, we asked about other NGO interventions and are only aware of a water, sanitation, and hygiene intervention in one of our sample communities; however, messages from mass media may have reached households in communities without specific interventions. The channels for such positive spillovers to noncash transfer beneficiaries include both attendance of nonparticipants at the training sessions and diffusion of nutrition related messages through informal networks. We have evidence that at least the first of these channels was active as even women who self‐reported as nonparticipants in the Cash for Nutrition programme attended the nutritional education sessions. Among self‐reported participants, the probability of attending at least one session during this period was on average 95.8%, but attendance among self‐reported nonparticipants was a surprisingly high 35.4%. An even higher percentage of nonparticipants (44%) acknowledged having learned new information from community educators. The second channel, diffusion through informal networks, does not have direct evidence in our data but would be consistent with findings from a recent randomized control trial to evaluate BCC interventions on feeding knowledge and practice in Bangladesh that found exposure to neighbour receiving a nutrition BCC information increased knowledge and feeding practices of nonparticipant mothers (Hoddinott, Ahmed, Ahmed, & Roy, 2017).

The existence of positive spillovers implies that because our identification strategy relies on comparing participants with non‐participants, the true impact on participants may be greater than our impact estimates show. The positive spillovers also point to the effectiveness of the community health volunteers in reaching women with health messaging without having to use the cash transfers as an incentive because the conditionality was not enforced. The soft conditionality approach allowed the programme to continue to reach even women who were temporarily displaced by the conflict.

In spite of being uniquely placed in a situation that allowed a CRT in the midst of a conflict situation, the study has some limitations. First, our results for practices are limited to self‐reports, so they may be affected by courtesy bias with mothers who have learned correct practices, reporting that they engaged in them to please the interviewer. That could lead to overestimation of programme impacts on practices.

On the other hand, some factors hindered our ability to measure programme impacts. The baseline scores for knowledge were high, which may have reflected the ease of guessing at the right answer in true/false questions used in our survey. Additionally, in some communities, baseline data could not be collected until after community health volunteers had already held the first training session. Exposure to this initial training by some participants likely explains the significant differences in knowledge between control and treatment groups at baseline and would also bias our impact estimates downwards.

Finally, our results refer to short‐term effects with the follow‐up survey collected immediately after the completion of the programme, so we are unable to provide evidence on sustainability of the impacts. Our results are also based on a CRT in specific to a particular programme and location in Yemen. In such cases, there is always room for concern about the external validity; however, our results certainly hold lessons for programs in similar contexts and are an important first step in understanding the effectiveness of BCC in humanitarian contexts given the lack of rigorous evidence currently available in this area.

Our findings demonstrate the effectiveness of culturally relevant BCC integrated with other interventions on modestly but meaningfully improving behaviour and knowledge, even in a context where implementation was subject to interruptions and the target population was under external stress. Our findings also suggest that although the use of primarily visual and interactive teaching styles can improve knowledge among illiterate women, this subpopulation may need to receive more intensive interventions than literate women to reach the same outcome levels.

## CONFLICTS OF INTEREST

The authors declare that they have no conflict of interest.

## CONTRIBUTIONS

SK designed the study. HI and SK conducted the data analysis. All authors contributed to the interpretations of the findings. JLF developed the first draft. SK wrote the final draft with contributions from all the authors.

## Supporting information

Data S1. Supporting information
