# Supplementary material for: Nutritional training in a humanitarian context: Evidence from a cluster randomized trial
Source: Matern Child Nutr. 2020 Mar 9;16(3):e12973. doi: 10.1111/mcn.12973 (PMC7296818; doi:10.1111/mcn.12973)
Supplement: Supplementary file 1 — Data S1. Supporting information [file MCN-16-e12973-s001.pdf]

Supplementary Tables

Figure A1. Summary of Knowledge Scores at Baseline and Expost in Treatment and Control Groups

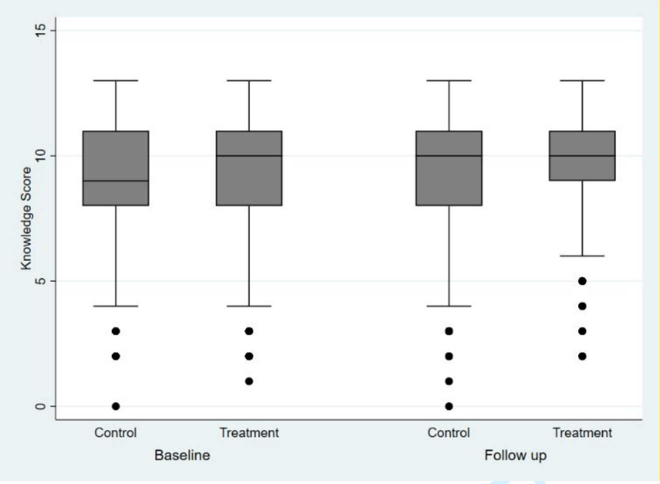

Table A1. EBF Robustness Checks.

	Randomized treatment	Randomized treatment	Self-reported treatment	Self-reported treatment
Cash for nutrition treatment	0.100*	0.0986*	0.125*	0.123*
	(0.0602)	(0.0588)	(0.0754)	(0.0735)
Change 2015-2017	0.00509	0.0280	-0.0151	0.00971
	(0.0511)	(0.0550)	(0.0566)	(0.0599)
Food distribution		-0.0632		-0.0643
		(0.0506)		(0.0501)
R-squared	0.0666	0.0805	0.0633	0.0724
N	252	249	252	249
Mean Dep Var	0.171	0.165	0.171	0.165
Village FE	Yes	Yes	Yes	Yes
Balance controls	Yes	Yes	Yes	Yes

1.Clustered standard errors at village level showed in parenthesis.
** $p < 0.10$, ** $p < 0.05$, *** $p < 0.01$*
